# Alzheimer Disease: Recent Updates on Apolipoprotein E and Gut Microbiome Mediation of Oxidative Stress, and Prospective Interventional Agents

**DOI:** 10.14336/AD.2021.0616

**Published:** 2022-02-01

**Authors:** Benson OA Botchway, Favour C Okoye, Yili Chen, William E Arthur, Marong Fang

**Affiliations:** ^1^Gastroenterology Department, Children's Hospital of Zhejiang University School of Medicine, National Clinical Research Center for Child Health, National Children’s Regional Medical Center, Hangzhou, China; ^2^College of Medicine, Zhejiang University, Hangzhou, China; ^3^Neurosurgery Department, Fourth Affiliated Hospital of Zhejiang University School of Medicine, Yiwu, China; ^4^Department of Internal Medicine, Eastern Regional Hospital, Koforidua, Ghana; ^5^Institute of Neuroscience, Zhejiang University School of Medicine, Hangzhou, China.

**Keywords:** Neurodegeneration, gut-brain axis, reactive oxygen species, antioxidants, mitochondria dysfunction

## Abstract

Alzheimer’s disease (AD) is a current public health challenge and will remain until the development of an effective intervention. However, developing an effective treatment for the disease requires a thorough understanding of its etiology, which is currently lacking. Although several studies have shown the association between oxidative damage and AD, only a few have clarified the specific mechanisms involved. Herein, we reviewed recent preclinical and clinical studies that indicated the significance of oxidative damage in AD, as well as potential antioxidants. Although several factors regulate oxidative stress in AD, we centered our investigation on apolipoprotein E and the gut microbiome. Apolipoprotein E, particularly apolipoprotein E-ε4, can impair the structural facets of the mitochondria. This, in turn, can minimize the mitochondrial functionality and result in the progressive build-up of free radicals, eventually leading to oxidative stress. Similarly, the gut microbiome can influence oxidative stress to a significant degree via its metabolite, trimethylamine N-oxide. Given the various roles of these two factors in modulating oxidative stress, we also discuss the possible relationship between them and provide future research directions.

## 1.Introduction

A progressive cognitive impairment is a significant indication of Alzheimer’s disease (hereafter: AD). AD has three main stages: preclinical AD, mild cognitive impairment, and dementia [1, 2]. In the United States, one out of every ten people aged 65 and up has AD [2]. The vast number of people affected with AD globally places a considerable strain on health resources. For example, as the disease progresses, patients become reliant on close family members, healthcare professionals, and caregivers to carry out their daily activities. The current medications for mild-to-moderate AD patients are cholinesterase inhibitors (galantamine, rivastigmine, and donepezil). Moderate-to-severe AD patients use memantine. Memantine suppresses the toxic effects of excess glutamate while simultaneously controlling its activation. Also, Namzaric (memantine + donepezil) is used to treat symptoms of moderate-to-severe AD. Unfortunately, current treatments merely deal with the symptoms, and medications are used cautiously due to their numerous adverse effects. Moreover, the efficacy of these medications decreases as the disease progresses [3].

Although enormous efforts have been undertaken to determine AD’s underlying causative and pathophysiological factors, these investigations have been largely inconclusive. However, recent studies such as the potentiality of 40-Hz light flickers as an interventional therapy and early detection of the disease via blood plasma have shown promising results [4, 5]. The latter study is particularly appealing because it evaluated potential disease biomarkers in a large number of cognitively impaired and unimpaired individuals, with their aberrancy associated with neurodegeneration [5]. Given the limited availability of biological indicators for monitoring AD development, blood plasma could be beneficial in studying the disease trajectory in both preclinical and clinical settings.

Mitochondria produce the ATP (adenosine triphosphate) needed for various physiological functions. Endoplasmic reticulum cytochrome P450, nicotinamide adenine dinucleotide phosphate-oxidase (NOX), and xanthine oxidase (XO) are among the mitochondrial and non-mitochondrial enzymes implicated in the endogenous free radical production via the electron transport chain [6]. Besides, the mitochondria regulate intracellular Ca^2+^ homeostasis and produce molecules that influence cellular apoptosis [7]. The inositol 1,4,5-triphosphate receptor (IP_3_R) regulates Ca^2+^ homeostasis and promotes increased intracellular Ca^2+^ by releasing Ca^2+^ from the endoplasmic reticulum (ER). As intracellular [Ca^2+^] levels rise, mitochondria absorb it through the intracellular mitochondria-associated membranes (MAMs). Also, Ca^2+^ exchange between the ER and mitochondria modulates mitochondrial respiration [8, 9], with its aftereffect being ROS (reactive oxygen species) formation [10].

Reactive oxygen species (ROS) are unstable, highly reactive species formed predominantly in the cell’s mitochondria as by-products of cellular metabolism. Oxygen-derived ROS include reactive nitrogen species (RNS), hydroxyl radicals, and superoxide anions. Hydrogen peroxide (H_2_O_2_) is an example of non-free radical ROS [11]. ROS (such as H_2_O_2_) are essential for cellular signaling in moderate quantities. For instance, low amounts of H_2_O_2_ promote cell growth in cancer and non-transformed stem cells [12]. Moreover, H_2_O_2_ is a second messenger that modulates macrophagic functions during the respiratory burst, which is notable in inflammation [13]. Nonetheless, chronic inflammation causes the overproduction of free radical species and leads to oxidative stress, impairing the neuronal membrane [14, 15].

ROS can be produced both exogenously (through medicines) and endogenously by the brain. The thioredoxin (Trx) system, which includes thioredoxin reductase (TrxR) and nicotinamide adenine dinucleotide phosphate hydrogen (NADPH), is essential for regulating ROS levels in the brain. The counteractive measures of the TrxR system in controlling ROS may be due to the minimal catalase expression in the brain [16]. Catalase is an antioxidant that can neutralize aberrant amounts of ROS to prevent oxidative stress. The brain’s reduced catalase is unsurprising, given that it requires high glucose metabolism and oxygen consumption for physiological activities. Thus, it is predisposed to oxidative stress due to higher ROS levels concomitant with limited counter-regulatory measures. In addition, biomolecules such as lipids are copious in the brain, increasing their susceptibility to oxidation in the presence of ROS [17].

The fundamental pathological features of AD comprise accumulated β-amyloid and tau protein phosphorylation. β-amyloid, in particular, causes neuronal damage and oxidative stress by disrupting the oxidant-antioxidant system, reducing synaptic plasticity, impairing metabolism, and inducing neuroinflammation [18]. On the other hand, an *in-vitro* study showed oxidative stress to aggravate soluble β-amyloid precursor protein and coincided with increased vascular endothelial growth factor (VEGF) [19].

Suggestions that a combination of several factors may cause AD have been proposed. Therefore, we conducted a systematic review to ascertain the role of apolipoprotein E (APOE) and gut microbiome dyshomeostasis in oxidative stress in AD. Also, we examined several potential antioxidants currently being explored for AD. Although the various sections of our theme are narrow, we aim to shed light and expand the horizon on the current understanding of the relationship between APOE, gut microbiome, and oxidative damage in AD.

## 2.Methods

### 2.1. Data sources and search terms

Publication databases and academic search engines, such as PubMed, Web of Science, and Scopus, were scoured extensively for studies used in this report. We conducted the searches at two different times. Two independent reviewers performed the first search (Benson and Favour) between July and August 2020. One independent reviewer (William) did the second search in January 2021. Search terms used were “Alzheimer disease” or “Neuro-degenerative disease” or “Neurodegeneration” and “oxidative stress” or “oxidative damage” or “gut microbiome oxidative stress” or “gut microbiome Alzheimer’s disease” or “gut microbiome neuro-degeneration” or “APOE and gut microbiome”. In addition, we narrowed our search to publications in English, with great emphasis on preclinical and clinical studies published within the last five years. Also, we prioritized *in*-vivo investigations over *in-vitro*.

In section 6, we used the clinicaltrials.gov database with the search terms “antioxidants for Alzheimer disease” together with filters “completed” (under ‘Status’), “studies with results” (under ‘Study Results’), and “approved for marketing” (under ‘Expanded Access below Status’) that yielded eight results. After, we amended the search terms to include studies that were currently in the recruiting stages. Thus, the search terms “antioxidant” and “recruiting studies” (under status) and “Alzheimer’s disease” generated ten studies.

### 2.2. Selection criteria

To include an article in the review, we used three indicators: 1) the study had to be original, along with the employment of appropriate animal models (either transgenic or drug-induced) and human samples; 2) the study had to involve both control and experimental groups; and 3) the study had to address the pathophysiological alterations (either β-amyloid plaques or neurofibrillary tangles), together with oxidative stress biomarkers. We omitted studies having the following criteria: 1) review articles; 2) non-experimental studies; 3) those with unavailable full texts, and 4) studies published over a decade ago. We consulted one reviewer (Yili or Marong) to discuss any disputes regarding a study inclusion or exclusion (supplementary Fig. 1).

## 3.Mitochondrial dysfunction and oxidative stress

Several factors contribute to mitochondrial dysfunction (MtD). Sirtuins (SIRTs), specifically SIRT3, are one example. SIRT3, abundant in the brain, is associated with mitochondrial function, aging, and longevity. Moreover, they regulate glutathione (GSH) [20]. Mitochondrial complex 1, which contains mitochondrial-NADH dehydrogenases 2/4 (MT-ND2 and MT-ND4), controls intracellular ROS levels. Reduced SIRT3 activity resulted in downregulated MT-ND2 and MT-ND4 levels, with concomitant effects of ROS overproduction, cytochrome c release, and neurodegeneration [21]. Cytochrome C is a pro-apoptotic factor, and studies have thoroughly investigated and reviewed the association between apoptosis and neurodegeneration [22-24]. Furthermore, recent studies have shown that elevated Ca^2+^ levels within the mitochondria cause neuronal death via free radical formation [25-26]. These findings are intriguing because Ca^2+^ accumulation in the mitochondria induces cytochrome c release into the cytosol via a stimulated mitochondrial-permeability transition pore (mPTP), with cytochrome c activating the caspase cascade to promote apoptosis [8-9]. Moreover, oxidative stress causes continuous alterations in the mitochondrial structure. For instance, prolonged oxidative stress disrupts functional tubular mitochondrial networks, resulting in mitochondrial fragmentation and apoptosis, decreased ATP production, energy metabolism, and an inability to meet neuronal cell energy demands [7]. Neuronal death occurs as a result of the limited availability of energy.

In the same way that mitochondria generate ROS, they also have mechanisms to handle ROS formation. For example, GSH peroxidase (GPx) is a free radical scavenger that regulates H_2_O_2_ within the mitochondria [27]. Similarly, copper-zinc superoxide dismutase 1 (SOD1) and copper chaperone for SOD1 (CCS) neutralizes mitochondria-generated free radicals [28]. However, damage to these mitochondrial antioxidative mechanisms might result in free radical aberrancy, and eventually, oxidative stress. For example, in the Haber-Weiss reaction, sustained elevated H_2_O_2_ levels within the mitochondria react with superoxide ions to form an even more reactive hydroxyl radical. In such an event, the cell’s fate is oxidative insults to proteins, mitochondrial DNA, and mPTP activation, which results in MtD and eventual cell death [13, 29].

mPTP appears to be a vital element in MtD, yet its structure and components are not well understood. However, we speculate that mPTP activation caused by either Ca^2+^ overload, stress, or unknown factors may result in the release of antioxidant systems from the mitochondria. Because mitochondria are the primary source of free radical production, the limited number of antioxidants that remain will be gradually overwhelmed by the constant formation of free radicals, ultimately causing oxidative stress and expediting related diseases, such as AD.

### 3.1.*Mitochondrial dysfunction, oxidative stress, and Alzheimer's disease*

Oxidative stress due to MtD has been implicated in AD, given that MtD is the chief instigator of free radical formation. The loss of mitochondrial function affects APP expression and processing, resulting in β-amyloid accumulation [30]. The build-up of intracellular β-amyloid in the mitochondria leads to its dysfunction. Furthermore, β-amyloid interaction with various mitochondrial proteins, the inner mitochondrial membrane, and the matrix impairs oxidative phosphorylation, increasing ROS synthesis [31]. The observed early manifestation of oxidative stress in AD could underlie its role in the disease etiology [17, 32]. Increased β-amyloid levels have been long linked to high degrees of oxidative proteins, lipids, and nucleic acids in the AD hippocampus and cortex [33]. Moreover, there is a relationship between AD and nuclear and mitochondrial DNA oxidation, as higher oxidized base (such as 8-hydroxyguanine and 5-hydroxyuracil) levels have been observed in the frontal, parietal, and temporal lobes of the brain [34-35]. Besides, a report indicated an elevated 8-hydroxyguanine in the hippocampus of preclinical stage AD patients [36].

MtD and tau pathology have a positive feedback interaction. The delivery of mitochondria in neurons is critical due to the importance of the mitochondria in maintaining neuronal function. Microtubule-associated proteins, like tau, transport mitochondria across the axon into synapses [37]. Overexpression and tau hyper-phosphorylation affect its localization and distribution [38, 39], impairing axonal transport, and causing synaptic loss [37]. Furthermore, hyperphosphorylated tau undermines mitochondrial function by lowering antioxidant activity. The resultant effect of this is oxidative stress, which hinders ATP production and causes synaptic dysfunction [40]. A preclinical investigation showed MtD to result from apoptosis, oxidative stress and upregulated voltage-dependent anion channel 1 protein (VDAC1). Interestingly, an upregulated VDAC1 was also noted in post-mortem AD brains [41], indicating that it may play a role in MtD. The question as to whether prospective VDAC1 inhibitors could alleviate MtD in AD is a future research topic that warrants thorough investigations using multiple preclinical models of AD.

## 4.Apolipoprotein E: Function and role in AD

Cholesterol is an organic molecule that is required for life sustenance and is generated chiefly in the liver. It maintains cell wall integrity and acts as a building block for many hormones (such as testosterone, cortisol, and aldosterone). Lipoproteins comprise apolipoprotein and lipids and transport cholesterol through the bloodstream [1, 42]. Apolipoproteins include apolipoprotein A-1 (APOA-I), apolipoprotein B-48 (APOB-48), apolipoprotein C-II (APOC-II), and apolipoprotein E (APOE). Their differences correspond to diverse molecular weights and functions [42]. For instance, APOC-II functions in energy delivery and storage through its hydrolyzation of chylomicrons and very-low-density lipoprotein (VLDL) [43]. We have previously reported the multiple polymorphic alleles of APOE and their frequency in humans [1]. APOE-ε4, in particular, has been associated with late-onset AD [44-46]. Furthermore, several cardiovascular and neurological diseases have shown the involvement of APOE [47-50]. The different APOE confer variable risks to AD. Although the precise reason for this variation has yet to be determined, several studies have attempted to provide some clarifications. These clarifications span from dysfunctions of the insulin signaling and blood-brain barrier (BBB) to levels of APOE concentration, cholesterol, and β-amyloid secretion [51-57]. For example, Chan and colleagues discovered that APOEε4-APP mice have much higher hippocampal β-amyloid levels than APOE-ε3-APP mice, which correlated with impaired insulin signaling and poor spatial memory [53]. The impaired insulin signaling pathway could be related to IGF-1 (insulin-like growth factor 1), IDE (insulin-degrading enzyme), and GLUT-4 (Glucose transporter type 4) reduced in significant amounts in the brains of animals carrying the APOE-ε4 allele [54]. Furthermore, Shinohara and colleagues discovered that APOE-ε2-TR mice have higher APOE in their CSF, cortex, hippocampus, and plasma than APOE-ε3 & ε4-TR mice. When cholesterol was measured, the CSF and plasma showed a similar pattern, and the brain cortex exhibited opposing results. The previous findings resulted in better memory function in APOE-ε2-TR mice than their age-matched counterparts [52]. Thus, there is the likelihood that higher APOE concentrations in AD might require a protective functioning gene (like APOE-ε2) to control brain cholesterol effectively and improve cognition, which may reduce AD risk. The findings from Shinohara et al. study [52] are supported by a recent study that examined over 5000 post-mortem brain tissues and reported a lower risk of homozygous APOE-ε2 people developing AD [58].

The BBB impermeability acts as a protective mechanism for the brain, preventing circulating bacteria and viruses from entering the brain. In a neurological condition, this protective mechanism may hinder therapeutic drug delivery for favorable consequences. Hence, the BBB mechanism is a double-edged sword. Dysregulated BBB in AD and other neurological diseases has been widely studied [55-57]. Particularly in AD, the involvement of the APOE-ε4 allele in BBB impairment has been evidenced, with the stimulation of the CypA-MMP9 signaling pathway touted as the potential mediator [59]. Therefore, future studies targeting this signaling pathway as an early interventional approach in halting or reversing cognitive dysfunction in AD might be worthwhile.

Sortilin is an important APOE metabolic regulator, and its role in AD is ambiguous. For instance, the interplay of APOE-ε3 and sortilin resulted in the modulation of omega-3 lipids. This finding corresponded with minimized levels of pro-inflammatory (TNF-α) and astrocytic (GFAP) markers. Not surprising, the opposite was true in APOE-ε4’s interaction with sortilin [60]. Furthermore, several human investigations have found disparate single nucleotide polymorphisms of the SORT1 (sortilin 1) gene; for example, rs17646665 lowers AD risk, while rs1010159 appears to increase the risk of mild cognitive impairment (MCI) progressing to AD [61, 62]. Notwithstanding, the SNP rs1764665 finding from the above study should be interpreted with caution due to its lack of correlation with conventional AD biomarkers, like β-amyloid and phosphorylated tau in cerebrospinal fluid (CSF) [62]. More intriguingly, previous studies also found no link between this genetic mutation and AD [63, 64]. Thus, SNP rs1764665 lower risk of AD may be relevant to those who live in a specific region, specifically Scandinavia, and maybe Europe.

### 4.1.Apolipoprotein E and oxidative stress

Several reasons explain the link between APOE and oxidative stress, one being APOE’s unique thiol-mediated antioxidant actions. Thiols, such as cysteine and GSH, are free radical scavengers with well-studied functional roles [65, 66]. Thiol levels are significantly reduced in AD [67, 68], leading to disease instigation or exacerbation. While APOE-ε3 and APOE-ε2 have corresponding one and two thiol-free groups, APOE-ε4 has none. The thiol groups of APOE-ε2 occupy positions 112 and 158 of the N-terminus. Likewise, that of APOE-ε3 is at 112 of the N-terminus [69]. Because the number of thiol groups corresponds to its antioxidant capabilities, a compound with more free thiol groups will have more robust antioxidant activity. On that basis, the absence of thiol-mediated activities in APOE-ε4 might compel the oxidation of biomolecules and enhance β-amyloid aggregation [70]. Similarly, the presence of two free thiol groups in APOE-ε2 may underlie its protectiveness in AD.

Superoxide dismutase (SOD) is a potent antioxidant enzyme that comes in three types: SOD1, SOD2, and SOD3. SODs - 1, 2, and 3 are in the cytoplasm, mitochondria, and extracellular space, respectively, and have varying physiological roles. A study demonstrated a positive relationship between SOD level and longevity in species like *Schizosaccharomyces pombe* and *Lasius niger* [71, 72]. Several studies have reported dysregulated SOD enzymes in both MCI and AD. Various biological markers, such as advanced oxidation protein products (AOPP) and ferric reducing antioxidant power (FRAP), are used to assess the degree of oxidative insults in both preclinical and clinical studies. Chico and colleagues evaluated the oxidative damage in 119 patients with MCI and AD. Relative to healthy controls, FRAP was considerably lower in MCI and AD subjects. Contrarily, AOPP was detected in more significant quantities in patient groups than in healthy controls. When comparing the SOD levels of MCI and healthy patients, MCI patients showed an increased level [73]. Several factors could have accounted for the increased SOD in MCI patients. For instance, both patient and healthy groups were on the Mediterranean diet, and the patient group might have adhered to the plan better than their healthy counterparts. However, the more significant finding was that 74% of the MCI patients were non-APOE-ε4 carriers. The importance of this discovery is because the increased SOD level in MCI patients relative to healthy controls might reflect the higher number of non-APOE-ε4 carriers. As to whether this result would have changed as the disease progressed is anyone’s guess.

Several studies have implicated FoxO (forkhead box O) and PCG-1α (peroxisome proliferator-activated receptor gamma coactivator 1-alpha) in various diseases, such as AD and oxidative stress disorders [74-76]. The involvement of FoxO-3 in AD or oxidative damage is puzzling due to studies showing contrasting results. For example, impaired HDAC (histone deacetylase) caused FoxO-3α upregulation, resulting in oxidative stress inhibition [77]. Similarly, the beneficial effects of FoxO-3 in obviating axonal dysfunction and oxidative insult have been evidenced [74, 75]. However, the initial findings are complicated by a study that found FoxO-3α to cause neuronal apoptosis via impaired miR-132/212, with concomitant effect of neurodegeneration [78]. Junxiang et al. did a post-mortem brain tissue analysis of APOE-ε4 carriers and non-carriers. Compared to non-APOE-ε4 carriers, they found that APOE-ε4 carriers had impaired mitochondrial structure and function due to mitigated levels of PCG-1α and SIRT3. Moreover, the observed mitochondrial dysfunction coincided with synaptic loss and oxidative stress [79]. PCG-1α is closely related to SIRT3, and both are essential modulators of mitochondrial dynamics. Their disparate functions have been thoroughly examined [76, 80-82]. During physical activities, the levels of these proteins are elevated [83, 84], and their overexpression alleviates mitochondrial detriments [76, 80]. These findings are interesting because they may underlie the rationale behind the clinical trial study by the University of Calgary (elaborated in section 6) to determine the possibility of physical activities in preventing neurodegeneration.

We have previously stated the relationship between APOE and sortilin. Sortilin-related vacuolar protein sorting 10 (VSP10P) domain-containing receptor 2 (SorCS2) belongs to the same transmembrane protein family as SORT1 [85]. Malik et al. showed SorCS2 upregulation to obviate neuronal death and oxidative injury by promoting neuronal cysteine. The increase in neuronal cysteine was concomitant with enhanced GSH formation. Most importantly, the interplay between SorCS2 and the excitatory amino acid transporter 3 (EAAT3) mediated the previously mentioned findings [86]. In AD, this transporter is abnormally aggregated in the hippocampus and causes neuronal decline [87]. Based on the prior studies, SorCS2 inhibition may impair EAAT3 functionality, trigger oxidative stress, and cause neuronal loss.

Although SODs are potent antioxidants, structural changes can reduce their efficacy. For example, rs2070424 and rs4880 are corresponding SOD1 and SOD2 genetic polymorphisms that increase AD risk [88, 89]. In individuals possessing combined APOE-ε4 and rs4880-T alleles, the likelihood of developing amnestic MCI (aMCI) was greater than in healthy controls, and the probability of getting AD was higher when compared to aMCI individuals [90]. However, in the absence of the APOE-ε4 allele, there was no risk of developing either AD or aMCI [90], implying that having the rs-4880 allele alone is not an AD risk. It is worth noting that several investigations [88, 91] have corroborated the previous finding. Therefore, we speculate that having the APOE-ε4 allele may cause antioxidant dysfunctionality of the SOD gene, especially in those carrying the rs-4880 variant, and increase their risk of AD.

Given that MCI is an antecedent condition to AD, alterations in oxidative stress biomarkers may indicate the progression of MCI to AD. For instance, the recent study by Deng et al. reported decreased SOD activity and increased plasma protein-methionine sulfoxide in AD individuals than MCI patients, indicating that oxidative stress was more profound in AD [92]. However, drawing a definitive conclusion will require further studies.

In summary, APOE-ε4 plays a multifaceted role in oxidative stress. We hypothesize that its modulation of SOD activity may result in; 1) the disease progression from MCI to AD, and 2) the activation of oxidative stress and other AD-related pathological features, which may exacerbate the disease. The careful monitoring of conventional oxidative stress biomarkers (such as AOPP and FRAP), coupled with interventions targeting their dysregulation may potentially alter the disease course from MCI to AD.

## 5.The gut microbiome

Microbial species abound and are diverse in the human gastrointestinal system. The majority of these organisms are bacteria, fungi, and viruses. An evolving body of knowledge concerning the gut microbiome and its effects on human health continues to grow. The human gut microbiota influences brain function via immunological, humoral, and neurological pathways [93]. Each person has an extensive gut microbiota that can be affected by diet and lifestyle, making the gut microbiota more susceptible to dysfunctional changes [94].

In this section, we review the possible link between the gut microbiome, APOE, and neurodegeneration. We also examine the gut metabolite involved in oxidative stress.

### 5.1.The gut microbiome and Apolipoprotein E

Multitudinous bacteria harbor within the gut. It is therefore not surprising that studies have associated several of them with AD. For instance, investigations have linked *Ruminococcaceae and Erysipelotrichaceae* with APOE. *Erysipelotrichaceae* and *Ruminococcaceae,* the most dominant bacteria families in the gut microbiome, are prevalent in AD patients [95, 96]. Parikh et al. found a correlation between the increased abundance of *Erysipelotrichaceae* in 4 and 6 - month-old EFAD mice and APOE-ε4 [95]. Also, an increase in *Erysipelotrichaceae* species was reported in 18-month-old APOE-ε4 transgenic mice relative to age-matched APOE-ε3 counterparts [96]. The proliferated population of this bacteria family in the gut microbiome appears to be related to higher cholesterol levels [97]. In addition, there is a positive correlation between this bacteria family and *TNF-α* [98]. Although the relationship between TNF-α and cholesterol concerning *Erysipelotrichaceae* is unclear, a high cholesterol diet may likely cause an aggravated formation of *Erysipelotrichaceae* species within the gut, which in turn could enhance TNF-α production and lead to inflammation [99]. It is worth mentioning that *APOE-ε*4 has a predilection for binding to VLDL and causes its build-up [100]. Hence, the *APOE-ε*4 transgenic mice used in [95] and [96] studies may have had elevated cholesterol levels caused by several factors, such as diet, lack of exercise, or both. The elevated cholesterol levels may have then triggered the increased Erysipelotrichaceae. Tran and colleagues also found considerably higher *Erysipelotrichaceae* in 4-month-old APOE-ε3 transgenic mice than in the age-matched APOE-ε4 transgenic mice group, although the reverse was true in 18-month-old animals [96]. Several factors, such as different animal breeds, different purchasing vendors, and ages of mice, may have accounted for the discrepancy in results between the two studies [95, 96] concerning the young-aged animals.

Compared to both APOE-ε3 and APOE-ε4 groups of similar age, a significantly high number of *Ruminococcaceae* species were discerned in APOE-ε2 EFAD mice [95]. Furthermore, disease-free individuals showed comparable findings [96]. *Ruminococcaceae* species produces short-chain fatty acids (SCFA), such as butyrate [96]. The beneficial effects of butyrate include the inhibition of β-amyloid plaque and controlling inflammation [101, 102]. In major depression and Crohn’s disease, reduced levels of these species have been observed [103, 104]. Particularly in Crohn’s disease, the reduced quantity of these species may have enhanced inflammatory factors and exacerbated the condition. Because APOE-ε2 is a protective allele in AD, we believe its protectiveness could be mediated by the proliferation of the *Ruminococcaceae* species in the gastrointestinal tract. Although the underlying reason is speculative, SCFA (like butyrate) generated by *Ruminococcaceae* specie*s* may modulate inflammation and minimize AD risk. Noteworthy is that a study found an association between the inhibition of the *NF-κB pathway and Ruminococcaceae* specie*s* [105], which could indicate the potential signaling pathway by which these bacteria control inflammation.

There is a link between APOE and the gut microbiome. Nevertheless, how APOE modulates the gut microbiota is presently unknown. Several hypotheses indicate inflammation as a central facet [95, 96], and APOE-ε4 is associated with pro-inflammation [106, 107]. This might explain why butyrate-generating gut bacteria, which can regulate inflammation, are considerably lower in APOE-ε4 than in APOE-ε2 alleles [95, 96]. Intriguingly, whether the increased relative abundance of butyrate-producing gut bacteria (via fecal transplantation or probiotics) in homozygous or heterozygous APOE-ε4 individuals could decrease AD risk, halt AD progression, or repudiate AD remains to be elucidated. Future studies delineating the above quandary with relevant animal models and human samples could improve our understanding and open novel interventional avenues for AD.

### 5.2.*The gut microbiome and neurodegeneration*

The correlation between the gut microbiome and neurodegeneration has generated significant interest in recent times and is an evolving investigative area. Sampson and colleagues demonstrated gut microbes to cause the augmentation of α-synuclein, a pathological feature of Parkinson’s disease. In addition, gut microbes generated short-chain fatty acids that resulted in neuroinflammation and affected the motor function of Thy1-α-Syn mice [108]. Preclinical and clinical AD investigations have evidenced alterations in the gut microbiome. For instance, the daily transfer of fecal microbiota in ADLP^APT^ (AD-like pathology with amyloid and neurofibrillary tangles) mice alleviated a myriad of AD-related pathological features and signs, such as β-amyloid accumulation, gliosis, tau pathology, and cognitive impairment. In addition, intestinal microbiota alterations aggravated the gut permeability, further leading to intestinal and systemic inflammation. Besides, these changes preceded AD pathogenesis [109]. The latter finding is interesting considering that a clinical study reported gut microbiota alteration preceding dementia [110]. Similarly, the evaluation of stool and inflammatory indicators showed increased inflammation in cognitively impaired individuals than aged-matched controls, concomitant with a higher abundance of proinflammatory taxa (*Escherichia/Shigella*). As expected, the anti-inflammatory marker (IL-10) was decreased in cognitively impaired individuals, also coinciding with a lower abundance of the anti-inflammatory taxon (*Eubacterium rectale*) [111].

**Figure 1.**
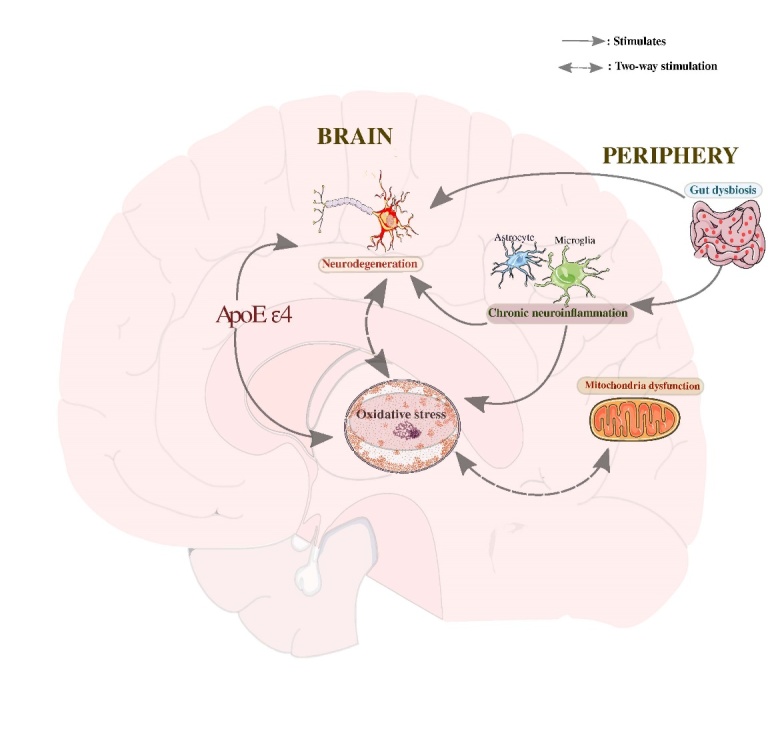
The various pathways resulting in oxidative stress and neurodegeneration. Lipopolysaccharides from gut dysbiosis impair the blood-brain barrier integrity, leading to neuroinflammation and neurodegeneration. During neuroinflammation, the activations of microglia and astrocytes enhance free radical synthesis that progressively increases the risk of oxidative stress. Furthermore, there is a higher risk of β-amyloid aggregation and oxidative stress in the absence of thiol-mediated antioxidant properties of APOE-ε4. β-amyloid plaques trigger neuronal damage and oxidative stress via the oxidant-antioxidant system disequilibrium. Chronic oxidative stress impairs mitochondrial function that exacerbates oxidative insults and causes neuronal dysfunction. Also, oxidative stress upregulates amyloid precursor protein, which may result in neurodegeneration. Relevant references are in the main text.

Given the above studies, peripheral factors may affect CNS disorders. We do concur with other studies [110, 112-114] in speculating that peripheral agent causing inflammation may disrupt the impermeable integrity of the BBB and gut. The resultant effect of this is the aggravation of harmful molecules (such as lipopolysaccharides; LPS) that could be passed through to the CNS to promote neuroinflammation and lead to neurodegeneration (Fig. 1).

### 5.3.*The gut microbiome and oxidative stress*

Investigations have established the correlation between gut microbiome dysbiosis and resultant health effects. A significant component of the pathway by which this occurs is oxidative stress. As has been outlined, ROS and RNS are crucial in body regulatory functions, not least are microbial destruction by neutrophils and cytokines. The inability of established counterregulatory factors, including GSH, to limit the deleterious effect of excess oxidative species on normal cell architecture has already been determined as the cause of many human ailments [115]. Regarding neurodegenerative diseases, studies have shown oxidative stress as a contributory factor. Gram-negative gut bacteria have been singled out as a possible etiological factor via the destructive effects of their LPS. A positive correlation exists between LPS and NADPH oxidase 2 (NOD2), with the potential resultant effect of neuroinflammation [114]. This is supported by several human studies that have ascertained the role of LPS in AD pathogenesis. For instance, examined brain tissues of AD patients revealed a higher level of LPS than age-matched healthy controls. More importantly, these observations were noted in amyloid plaques and surrounding blood vessels [116]. Also, human AD brains showed a higher degree of LPS in the hippocampal and superior temporal lobe [117]. Despite unclear reasons, one possible route by which LPS induces oxidative stress in AD could be through its concerted interaction with CD-14 (cluster of differentiation-14) and TLR-4 (toll-like receptor-4), which then triggers both astrocytes and microglia. The instigation of these cells may cause the release of complement proteins, proinflammatory cytokines, and chemokines, resulting in oxidative stress. Both mitochondria damage and chronic inflammation are consequences of oxidative stress that can eventually lead to neuronal loss [118].

Trimethylamine N-oxide (TMAO) is a gut metabolite, and studies have associated it with several human diseases. Salmon, milk, and red meat consumption in humans undergo initial metabolization into trimethylamine (TMA) by the gut bacteria. Following its absorption via the intestinal walls, TMA is then conveyed to the liver. With oxygenation, the liver breaks it down to TMAO [119]. In analyzing AD and MCI patients, their CSF showed higher levels of TMAO. Concomitantly, neuronal degeneration and other AD-related pathological markers, such as aberrancy in the ratio of phosphorylated tau to β-amyloid were also discerned [120]. Several human and animal studies have pointed to TMAO as being involved in oxidative stress, with its exacerbated levels associated with aging. In APOE knock-out mice put on a diet that included TMAO, increased plasma levels of TNF-α, MCP-1, and IL-1β (implying inflammation), as well as corresponding higher and lower plasma concentration of malondialdehyde and GSH-Px/SOD activities (indicating oxidative stress) were detected [121]. Also, TMAO level was higher in aged Fisher-344 male rats than in younger controls. The above observation concurred with the exacerbated formation of superoxide, upregulated pro-inflammatory cytokines, and downregulated endothelial nitric oxide synthase [122]. These findings are supported by a more recent that showed TMAO to instigate oxidative stress and cause endothelial impairment in older individuals [123]. However, a study has reported contrary findings concerning TMAO’s association with oxidative stress. Intriguingly, this study used only healthy women (between 65 and 70 years) and detected no association between TMAO and oxidative stress following a 24-week supplementation of 1500-mg L-carnitine-L-tartrate [124]. To our knowledge, this is the only study to report the absence of any link between TMAO and oxidative stress.

We suspect that increased aging, particularly in AD, may cause elevated circulatory levels of TMAO that can trigger the overexpression of cytokines to promote oxidative stress and compromise endothelial function, eventually resulting in neurodegeneration. Nonetheless, a more precise delineation of the association between TMAO and oxidative stress warrants further studies using applicable human and animal models. Most importantly, data analysis from these studies should factor in the age, sex, and disease state of experimental models. Also, when assessing TMAO in AD, neurofibrillary tangles and β-amyloid plaques should be considered. Similarly, as certain foods are sources of TMAO, there is the likelihood that significant moderation of these foods may be beneficial for aged individuals, especially those at risk of AD. The above statement will corroborate our previous report where we suggested nutrition as a possible interventional agent for AD [125].

Complex interactions between microbiota, as previously reported, influence oxidative species and hinders the antioxidant counterregulatory mechanisms. Various metabolites of microbiota, including short-chain fatty acids, TMAO, and polyphenols, may regulate the oxidative state of the CNS.

## 6.Prospective interventional agents

AD’s complex pathophysiological mechanism partly accounts for the lack of an effective treatment. For almost two decades, no pharmaceutical agent had been approved to either reverse or improve disease symptoms until September 2019, when health regulators in China endorsed the use of sodium oligomannate for mild-to-moderate AD. Before its endorsement, Xinyu and colleagues used human and animal models (5xFAD (Tg), C57, and APP/PS1 mice), where they found sodium oligomannate to alter the gut microbiota, with concomitant effects of attenuated neuroinflammation and enhanced cognition [126]. The significance of this study lies in shedding light on the possibility that targeting the gut microbiota dysregulation and its metabolites could halt the progression of AD-related pathological indicators and ameliorate the disease.

Antioxidants, classified as endogenous (primarily enzymes) or exogenous (non-enzymes obtained from food products), can halt oxidative stress. The endogenous antioxidant includes SOD, catalase, and GSH. The GSH system comprises GSH peroxidase (GPx) and GSH reductase [127]. Given that antioxidants counteract the activity of free radicals, a thorough understanding of their usefulness for AD and other neurodegenerative diseases is paramount. Catalase and GPx convert H_2_O_2_ to less harmful substances (water and oxygen). However, the GPx-GSH system is limited by the availability of GSH, which has to be regenerated from GSSG (glutathione disulfide) by flavoenzyme, GSH reductase, and NADPH. This limitation decreases the efficiency of the GPx-GSH system in removing H_2_O_2_ [27]. Several antioxidants for AD have undergone clinical trials. For example, the use of resveratrol in mild-to-moderate AD patients showed increased and decreased plasma matrix metalloproteinase (MMP)-10 and CSF MMP-9, respectively. In addition, regulated neuroinflammation via elevated fibroblast growth factor-2, macrophage-derived chemokine, and IL-4, as well as reduced RANTES (Regulated on Activation, Normal T-Cell Expressed and Secreted) and IL12p40 was detected [128]. These findings corresponded with enhanced mini-mental status examination (MMSE) and Alzheimer's Disease Cooperative Study—Activities of Daily Living Inventory (ADCS-ADL) scores and reduced β-amyloid in the CSF. Concerning the inhibition of some ILs, particularly IL12p40, there have been suggestions that its impairment could reduce accumulated β-amyloid plaques while enhancing cognition [129, 130]. In phase 2 randomized controlled trial, the administration of 2-mg circadin (prolonged released melatonin) tablets showed considerable improvement in productive sleep and cognitive function. Noteworthy is that this medication was given along with anticholinesterase inhibitors, indicating the efficacy of a combinational therapy for AD. Besides ascertaining circadin’s potency, the study also showed the possible association between cognitive decline and sleep insufficiency [131]. Studies have evidenced the link between AD and sleep dysfunction. For instance, an 11.2-year observational study of non-demented subjects correlated premature light-out time and prolonged sleep latency with dementia risk, particularly AD. Premature light-out time and prolonged sleep latency are indicators of poor sleep quality [132]. Intriguingly, the study found the absence of a correlation between dementia risk and disruption to the 24-hr activity rhythm. This finding complicates the specific relationship between sleep disturbance and AD, given that rest-activity rhythm disturbance in AD has been linked to increased and decreased NREM (Non-rapid eye movement) sigma and delta power, respectively [133]. Regardless of these contrasting findings, the potential applicability of melatonin for AD as a preventive agent has given grounds for researchers from the University of Iowa to undertake a 9-month clinical trial study involving 230 participants. Study participants will be administered 5-mg melatonin and have their AD biological markers assessed at various time points. For instance, evaluations of CSF t-tau and the ratio of p-tau to β-amyloid will take place eight weeks before initiating treatment and weeks 16 and 44 after treatment. Also, neuropsychological examinations will be employed to evaluate cognition (*from clinicaltrials.gov, NCT03954899*). Aside from being antioxidants, melatonin and resveratrol are SIRT1 activators and can potentially mitigate AD pathological features. In particular, SIRT1’s role in AD has been extensively reviewed [134], implying that their applicability in AD could be promising. Therefore, it would be interesting if relevant AD models harboring both β-amyloid and tau pathological features can evaluate the efficacy of the combined usage of melatonin and resveratrol for the disease.

**Table 1:** Summary of clinical studies of prospective antioxidants for AD (Compiled from clinicaltrials.gov)

Clinical trial number	Interventional agents	Aim	Number of participants and age range (years)	Current status	Primary purpose	Estimated completion date
NCT03514875	MitoQ	To evaluate the impact of this agent on cerebrovascular blood flow and carotid artery vasodilation	N = 12 Age: 50-85	Recruiting	Treatment	October 2021
NCT03841539	Mediterranean diet Low fat diet	To ascertain the effect of these interventions on brain volume and antioxidant level, as well as memory and cardiometabolic biological markers	N = 200 Age: ≥ 65	Recruiting	Prevention	February 2023
NCT01982578	Genistein	To establish the efficacy of this agent in AD	N= 50 Age: ≥ 18	Recruiting	Treatment	December 2020
NCT03978052	Epigallocatechin-gallate plus physical activity plus diet plus mental health promotion	To determine whether this multimodal action can halt cognitive decline	N = 200 Age: 60-80	Recruiting	Prevention	September 2021
NCT04740580	Glutathione (Glycine plus N-acetylcysteine)	To assess the impact of glutathione on cognition	N = 52 Age: 55- 85	Recruiting	Other	May 2025
NCT03361410	Grape powder	To evaluate the impact of this agent on neuropsychological behavior and cerebral metabolism	N = 32 Age: 65 - 85	Recruiting	Treatment	January 2021
NCT04063124	Dasatinib plus Quercetin	To ascertain the penetrance ability of this combined agents in AD individuals	N = 5 Age: ≥ 65	Recruiting	Treatment	August 2023
NCT01780974	Lipoic acid plus omega-3 fatty acids	To evaluate the efficacy of this combined agent in inhibiting AD	N = 42 Age: ≥ 55	Completed	Prevention	October 2015
NCT01354444	Carvedilol	To determine if this agent can ameliorate AD	N = 29 Age: 0 - 100	Completed	Treatment	January 2017
NCT01058941	Lipoic acid plus fish oil concentrate	To ascertain if the combined agent can halt AD progression	N = 67 Age: ≥ 55	Completed	Treatment	December 2014
NCT00597376	Cerefolin NAC	To determine the correlation between this dietary supplement and cognition	N=104 Age: ≥ 60	Completed	Prevention	May 2011

MitoQ: mitoquinone; AD: Alzheimer disease

Vitamins can alleviate oxidative stress and modulate inflammation by neutralizing free radical activities, thus being potential adjuncts in AD treatment [125]. Compared to the placebo-administered group, a 5-year administration of α-tocopherol (vitamin E) showed remarkable retardation of activities of daily living in mild-to-moderate AD patients [135]. We noted two major factors from this study. Firstly, the study participants were already on ChEIs (donepezil and galantamine), suggesting that this study was ascertaining the effectiveness of a combinational therapy for AD. Secondly, α-tocopherol alone (i.e., α-tocopherol + ChEI) was more effective than the combined administration of α-tocopherol and memantine (i.e., α-tocopherol + memantine + ChEI). Contrastingly, a concluded clinical trial found the combined usage of omega-3 fatty acids and lipoic acid to decelerate both functional and cognitive deteriorations in AD patients [136]. We previously suggested the employment of combinational therapy as a probable intervention for AD [125]. As might be expected, along with the clinical evidence from this observational study [135], not all combinational therapies are likely to be effective. Nonetheless, we still believe that combined interventional agents will be the right course for AD due to the intricate pathophysiological mechanism of the disease, with extensive studies needed for better understanding before the employment of this course of action.

Finding an effective interventional agent for AD looks more promising than ever owing to a greater understanding of the disease trajectory over the last decades since its first report by Alois Alzheimer, together with the development of innovative diagnostic techniques. Table 1 summarizes clinical studies on antioxidants that have either been completed or are in the recruiting stage. Paul Rosenberg and colleagues from John Hopkins University and Icahn School of Medicine are undertaking a phase 1 clinical trial that will employ bioactive dietary polyphenol preparation (BDPP, comprising resveratrol and grape seed polyphenolic extract) to ascertain its efficacy against prediabetes or type 2 diabetes and MCI. Diabetes is a known AD risk factor [137, 138]. Assessed outcomes will include cognition, mood, and adverse effects (*from clinicaltrials.gov, NCT02502253*). Also, Marc Poulin and colleagues will conduct a non-invasive clinical study on the use of aerobic exercise (walking and jogging) for individuals at high risk of AD and associated dementia. The study concludes in January 2025 and will assess brain structure and function, coupled with sleep quality and cognition at different time points. In essence, the study seeks to demonstrate the link between physical activity and cognitive processes and its potential in halting age-related neurodegeneration (*from clinicaltrials.gov, NCT03035851*).

## 7.Concluding remarks and research directions

The implication of oxidative stress in AD is multifactorial. However, whether it induces or is an aftereffect of AD, along with its precise time of emergence (either early or late stage of the disease), is presently open to question. There is the likelihood of oxidative stress being an instigator, accelerator, and aftermath of AD. Given that completed multitudinous AD studies are leaving us with unanswered queries, more thorough investigations need to address the specific position of oxidative stress in the pathophysiological context of AD, which could be instrumental in the development of effective interventions for the disease. Future studies on the subject of oxidative stress in AD could include:

• Identifying the “driver” and “passenger” between β-amyloid and tau protein and oxidative damage. Is β-amyloid/tau protein the “driver” that spurs oxidative damage or vice versa?
• Determining various gut bacteria family/phylum species involved in oxidative stress and their regulatory targets.
• Thorough elucidation of oxidized lipids and proteins that may serve as AD biomarkers in the clinical setting.
• Targeting oxidative damage in the early AD stage via gut microbiome modulation and ascertaining its likelihood of halting disease progression
• Establishing the specific role of cell cycle dysfunction in AD and discovering innovative approaches to repairing this dysfunction.
• Determining the specific features of mitochondria involved in AD and their potential targets for prospective pharmaceutical agents.
• Establishing the possible relationship between mammalian vacuolar protein sorting 10 protein, APOE, and oxidative stress.

## Supplementary Materials

The Supplementary data can be found online at: www.aginganddisease.org/EN/10.14336/AD.2021.0616.


