# Supplementary material for: Alzheimer Disease: Recent Updates on Apolipoprotein E and Gut Microbiome Mediation of Oxidative Stress, and Prospective Interventional Agents
Source: Aging Dis. 2022 Feb 1;13(1):87–102. doi: 10.14336/AD.2021.0616 (PMC8782546; doi:10.14336/AD.2021.0616)
Supplement: Supplementary file 1 [file AD-13-1-87-s.pdf]

SUPPLEMENTARY DATA

# Alzheimer Disease: Recent Updates on Apolipoprotein E and Gut Microbiome Mediation of Oxidative Stress, and Prospective Interventional Agents

**Benson OA. Botchway[1,2,5], Favour C. Okoye[2], Yili Chen[3*], William E. Arthur[4], Marong Fang[1,5*]**

© 2021. Botchway B. et al. Published online at http://www.aginganddisease.org/EN/10.14336/AD.2021.0616

# SUPPLEMENTARY DATA

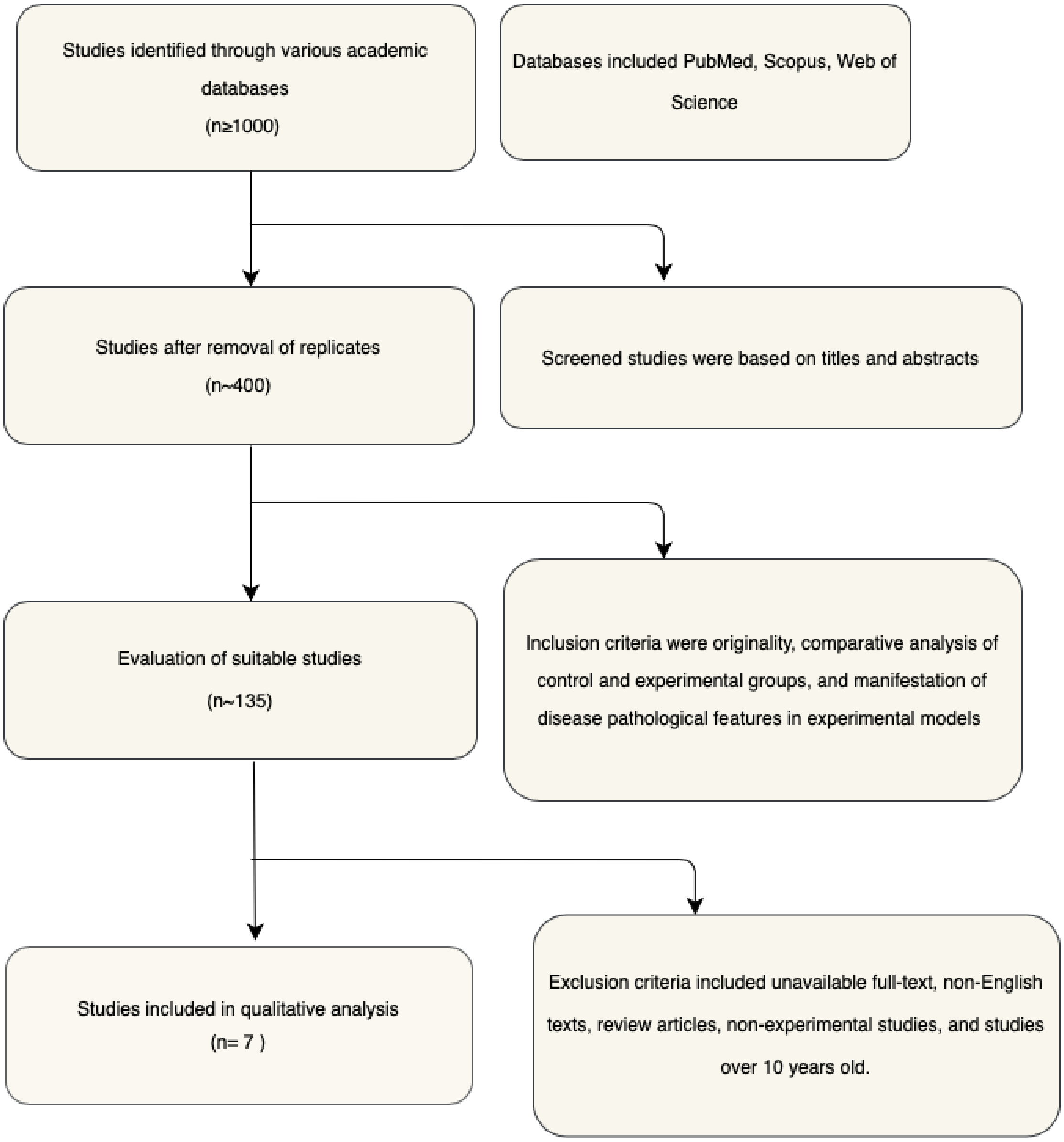


**Supplementary Figure 1. Flowchart for inclusion of studies examining apolipoprotein E and gut microbiome modulations of oxidative stress in Alzheimer's disease.**

© 2021. Botchway B. et al. Published online at http://www.aginganddisease.org/EN/10.14336/AD.2021.0616
