# Supplementary figures and images for: Alzheimer Disease: Recent Updates on Apolipoprotein E and Gut Microbiome Mediation of Oxidative Stress, and Prospective Interventional Agents
Source: Aging Dis. 2022 Feb 1;13(1):87–102. doi: 10.14336/AD.2021.0616 (PMC8782546; doi:10.14336/AD.2021.0616)

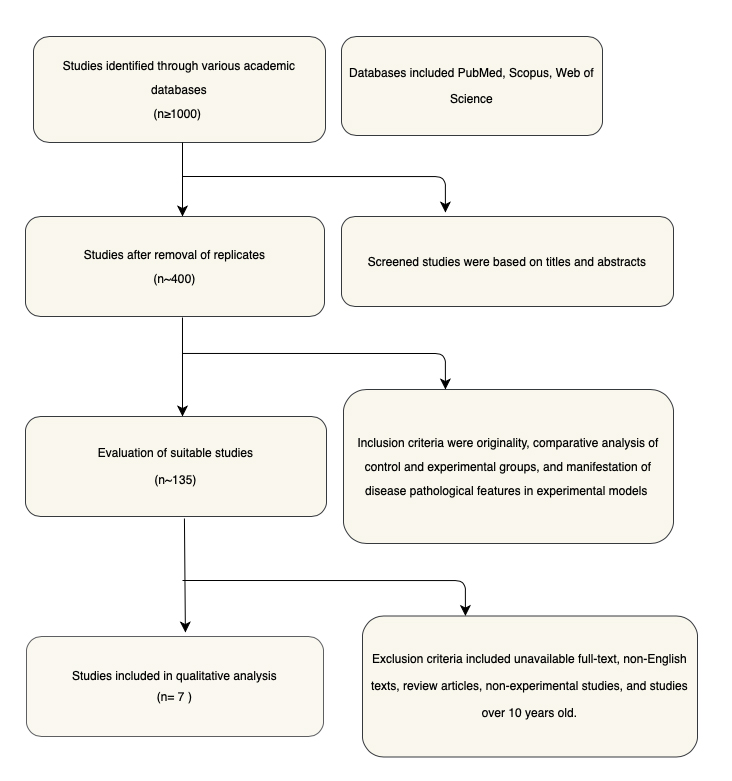

Supplement: Supplementary file 2 [file ad-13-1-87-s-g1.jpg]
